# Effects of 17β-oestradiol on rat detrusor smooth muscle contractility

**DOI:** 10.1113/expphysiol.2009.047118

**Published:** 2009-04-24

**Authors:** Aurora Valeri, Keith L Brain, John S Young, Giampietro Sgaragli, Federica Pessina

**Affiliations:** 1 Department of Neuroscience, Siena UniversitySiena, Italy; 2 University Department of Pharmacology, University of OxfordOxford, UK

## Abstract

The aim of this study was to investigate the effect of 17β-oestradiol (E_2_) on detrusor smooth muscle contractility and its possible neuroprotective role against ischaemic-like condition, which could arise during overactive bladder disease. The effect of E_2_ was investigated on rat detrusor muscle strips stimulated with carbachol, KCl and electrically, in the absence or presence of a selective oestrogen receptor antagonist (ICI 182,780) and, by using confocal Ca^2+^ imaging technique, measuring the amplitude (Δ*F*/*F*_0_) and the frequency of spontaneous whole cell Ca^2+^ flashes. Moreover, the effect of 1 and 2 h of anoxia–glucopenia and reperfusion (A-G/R), in the absence or presence of the hormone, was evaluated in rat detrusor strips perfused with Krebs solution which underwent electrical field stimulation to stimulate intrinsic nerves; the amplitude and the frequency of Ca^2+^ flashes were also measured. 17β-Oestradiol exhibited antispasmogenic activity assessed on detrusor strips depolarized with 60 mm KCl at two different Ca^2+^ concentrations. 17β-Oestradiol at the highest concentration tested (30 μm) significantly decreased detrusor contractions induced by all the stimuli applied. In addition, the amplitude and the frequency of spontaneous Ca^2+^ flashes were significantly decreased in the presence of E_2_ (10 and 30 μm) compared with control detrusor strips. In strips subjected to A-G/R, a significant increase in the amplitude of both spontaneous and evoked flashes was observed. 17β-Oestradiol was found to increase the recovery of detrusor strips subjected to A-G/R. The ability of E_2_ to suppress contraction in control conditions may explain its ability to aid recovery following A-G/R.

The urinary bladder functions to store and to expel the urine from the body. These roles are achieved through complex interactions between the autonomic nervous system, the sensory nerves and the urinary bladder smooth muscle. Normal bladder function depends on the integrity of these interactions and is maintained by an adequate supply of oxygen and nutrients via the circulation. Greenland *et al.* (2000) observed a period of ischaemia and hypoxia during normal micturition in pigs, and noted that partial bladder outlet obstruction increased the severity and duration of bladder wall hypoxia. The effects of hypoxia/anoxia have been studied in a variety of smooth muscles, and several mechanisms could potentially contribute to hypoxia-induced reduction in force. Such mechanisms fall in two categories. The first relates to energy limitation, viewing cellular ATP production under hypoxic conditions as being unable to support actin–myosin ATPase activity, hence, contractile activity (Obara *et al.* 1997). The second involves some form of oxygen sensing that subsequently leads to modulation of pathways involved in excitation–contraction coupling. Both Ca^2+^-dependent mechanisms, involving hypoxia-induced changes in intracellular [Ca^2+^], and Ca^2+^-independent mechanisms, involving the Ca^2+^ sensitivity of the contractile apparatus, may contribute (Shimizu *et al.* 2000). In addition to contractile dysfunction, ischaemic injury to the mucosa causes increased mucosal permeability and activation of sensory nerves with subsequent detrusor overactivity (Azadzoi *et al.* 1996), which may be related to irritability symptoms such as urgency, frequency and urge incontinence (the components of overactive bladder syndrome). Overactive bladder affects 33 million adults in the United States, which is approximately 16.5% of the population (Stewart *et al.* 2003). Knowledge of how the detrusor responds to ischaemic conditions is necessary for the development of ways to treat this syndrome.

Oestrogens are steroids, named for their importance in the oestrous cycle, which function as the primary female sex hormone. The most potent naturally occurring oestrogen in humans is 17β-oestradiol (E_2_). Oestrogens have widespread biological actions. They stimulate growth, blood flow and water retention in sexual organs and they also influence differentiation, maturation and function of various tissues throughout the body, including the peripheral and central nervous systems. Furthermore, oestrogens have been shown to have beneficial effects in cellular and molecular systems relevant to neurodegenerative disorders (Behl *et al.* 1997). 17β-Oestradiol is a vaso- and neuroprotective agent (Green & Simpkins, 2000; Roof & Hall, 2000). It has been demonstrated to inhibit lipid peroxidation and protects neurons against oxidative stress (Behl & Holsboer, 1999).

Recently, Pessina *et al.* (2007) have observed in guinea-pig urinary bladder that there is a higher resistance to the effects of anoxia–glucopenia and reperfusion (A-G/R) in females compared with males; it was argued that E_2_ might be responsible for this difference. Moreover, E_2_ might affect the intracellular Ca^2+^ concentration (Pozzo-Miller *et al.* 1999), reducing Ca^2+^ influx primarily through the inhibition of L-type Ca^2+^ channels in a non-genomic manner and therefore decreasing myosin light chain (MLC) phosphorylation and contraction of smooth muscle (Kitazawa *et al.* 1997). In addition, E_2_ could activate Ca^2+^-dependent molecules, such as protein kinase C and Ca^2+^–calmodulin (Hayashi *et al.* 1994; Kelly *et al.* 1999).

Oestrogens have been used for several years to treat urinary symptoms, especially those associated with the lower urinary tract. The action of oestrogen on the continence mechanism is likely to be complex. Oestrogens may affect continence by any of the following mechanisms: (a) increasing urethral resistance; (b) raising the sensory threshold of the bladder; (c) increasing α-adrenoreceptor sensitivity in the urethral smooth muscle; and (d) promoting β-adrenoceptor-mediated relaxation of the detrusor muscle (Kinn & Lindskog, 1988; Busby-Whitehead & Johnson, 1998; Matsubara *et al.* 2002). However, contradictory effects of oestrogens on bladder contractility have been reported (Diep & Constantinou, 1999; Jackson *et al.* 2002).

The aim of the present study was to investigate the effect of 17β-oestradiol on detrusor smooth muscle contractility and its possible role as neuroprotective agent against damage resulting from A-G/R. The effects of E_2_ on detrusor smooth muscle contraction were investigated using both contraction and confocal Ca^2+^ imaging.

## Methods

### Preparation of detrusor strips

All experiments were performed in strict compliance with the recommendations of the EEC (86/609/CEE) for the care and use of laboratory animals and were approved by the Animal Care and Ethics Committee of the University of Siena, Italy. Sixty Wistar male rats (Charles River, Calco, Italy; 250–400 g) were anaesthetized with a mixture of ketamine hydrochloride (30 mg kg^−1^, i.p; Ketavet®, Gellini, Aprilia, Italy) and xylazine hydrochloride (8 mg kg^−1^, i.p; Rompum®, Bayer, Wuppertal, Germany) and killed by cervical dislocation. The bladders were isolated, cleaned of external fat and connective tissue, and opened along the ventral surface. Strips of detrusor muscle measuring approximately 1.0 mm × 0.5 mm × 8 mm were dissected following the direction of the muscle bundles. Fine silk ligatures were tied to each end of the strips, which were mounted in small (0.2 ml) superfusion organ baths between two platinum electrodes 1 cm apart. Strips were continuously superfused with Krebs solution (composition in mm NaCl, 120; KCl, 5.9; MgCl_2_, 1.5; CaCl_2_, 2.5; NaHCO_3_, 15.4; NaH_2_PO_4_, 1; glucose, 11.5; pH 7.4) pumped by a peristaltic pump (Watson-Marlow, Falmouth, UK) at a constant rate of 1.5 ml min^−1^. Strips were placed under an initial tension of 10 mN and allowed to equilibrate for at least 60 min. Contractions were measured isometrically using mechanoelectrical transducers (Basile, Comerio, Italy) and recorded using a PowerLab 8/30 data acquisition system (ADInstruments, Basile, Comerio, Italy) connected to a notebook computer running Chart 5 software (ADInstruments). Electrical field stimulation (EFS; 0.05 ms pulse duration, 50 V, 10 Hz, in 5 s trains) was delivered via a digital stimulator (LE 12106, LETICA Scientific Instruments, Barcelona, Spain) every 30 min. From preliminary experiments, when tissues were pre-incubated with 3 μm TTX for 20 min, EFS responses were 4.7 ± 1.6% of control values (*n*= 4), demonstrating their neurogenic origin.

### The effects of E_2_ on strips subjected to ischaemia–reperfusion-like conditions (anoxia–glucopenia and reperfusion; A-G/R)

In order to mimic ischaemic conditions, a number of modifications were carried out to the organ bath apparatus. The Krebs solution at 37°C was replaced by a glucose-free Krebs solution (glucose was replaced isosmotically with NaCl; glucopenia) and the solution was gassed with 95% N_2_ and 5% CO_2_ (anoxia). After this A-G period, initial conditions were restored (reperfusion).

To test the oxygen tension in the bath during A-G conditions, oxygen was measured with a galvanic oxygen electrode (model MLT1115; ADInstruments, Chalgrove, UK). The electrode was calibrated using a three-point calibration with glucose-free Krebs solution (equilibrated in a large reservoir for 3 h with 0%O_2_–95%N_2_–5%CO_2_, air or 95%O_2_–5%CO_2_). The equivalent O_2_ saturation of the hypoxic–glucopenic solution in the contraction bath was 0.7%. Assuming an atmospheric pressure of 760 mmHg, this implies a partial pressure of O_2_ under anoxic–glucopenic conditions of 5 mmHg.

The response of intrinsic nerves to EFS was expressed as a percentage of the initial response in standard Krebs solution, taken as 100%.

In a first set of experiments, after a 60 min equilibration period, in which control responses to various stimuli were obtained, strips were subjected to 60 min of A-G conditions followed by 120 min of R. In a second set of experiments, the length of the A-G phase was extended to 120 min, followed by 180 min of R. Drugs (E_2_ at 0.1, 1, 3, 10 or 30 μm) were added to the superfusing solution 60 min before applying A-G (pre-incubation, P-I), during A-G and during the first 30 min of R, while stimulating the strips every 30 min, as described in the previous subsection.

### Effects of E_2_ on detrusor strip contractility

Detrusor strips, placed in the organ bath with standard Krebs solution at 37°C and bubbled with 95% O_2_–5% CO_2_ gas mixture, were left to equilibrate for 60 min_._ Then, each strip was stimulated in a random order: electrically (pulses of 0.05 ms, 50 V and 10 Hz in 5 s trains), with 10 μm carbachol (CCh) or with high-potassium (60 mm) solution with 30 min intervals between each stimulus. To examine the concentration-dependent effects of E_2_ on muscle contractility, each strip was exposed to a different concentration of the hormone (0.1, 1, 3, 10 or 30 μm) or to the solvent (ethanol), taken as control. After 20 min of incubation, strips were stimulated again in the presence of E_2_ or ethanol.

The involvement of oestrogen receptors (ERs) in the effects of E_2_ was assessed by incubating the strips for 20 min with the selective ER antagonist ICI 182,780 (at the same concentration of E_2_) before addition of E_2_ to the organ bath.

### Antispasmogenic activity of E_2_ on detrusor smooth muscle strips

The antispasmogenic effect of E_2_ or nifedipine was assessed in strips in which contractions were elicited by depolarization with 60 mm K^+^ in the presence of 0.5 or 5 mm Ca^2+^. Krebs solution containing 60 mm K^+^ was prepared by replacing NaCl with equimolar KCl. When the Ca^2+^ concentration was changed, Ca^2+^ was replaced isosmotically with NaCl. After a 60 min equilibration period with Krebs solution containing either 0.5 or 5 mm Ca^2+^, strips were exposed to high K^+^ for 4 min every 20 min, until responses were reproducible, and these were taken as control values. Drugs at increasing concentrations (E_2_ at 0.1, 1, 3, 10 and 30 μm; nifedipine at 0.1, 1, 10 and 100 nm and 1 μm) were tested on successive responses to high K^+^ (each compound on a different strip, repeated on strips from 4 animals). Drugs were applied 10 min before as well throughout the depolarizing period (Pessina *et al.* 2001). Results are expressed as percentage of inhibition with respect to control values. The pharmacological effect of each substance is described as the mean ±s.e.m. value of the pIC_50_.

### Calcium imaging and analysis

After subjecting tissues to ischaemic-like conditions, as previously described, each detrusor strip was exposed to 10 μm Oregon Green-488 1,2-bis(*O*-aminophenoxy) ethane-*N*,*N*,*N*′,*N*′-tetraacetic acid-1 acetoxymethyl ester (BAPTA-1 AM) in 1% dimethyl sulphoxide and 0.2% pluronic F-127 in standard Krebs solution for 90 min at 36°C. Each strip was then rinsed in Krebs solution, and bubbled with 95% O_2_ and 5% CO_2_ for at least 10 min. Tissues were then pinned flat, serosal side up, in a Sylgard®-lined organ bath and mounted on the stage of an upright confocal microscope.

The detrusor strips were continuously superfused with Krebs solution (bath temperature 33–34°C). Images were acquired with a Leica SP2 upright confocal microscope (Leica Microsystems, Milton Keynes, UK). Oregon Green-488 BAPTA-1 AM was excited with 488 nm laser light, and emission was collected through a prism and shutters set to pass wavelengths longer than 510 nm. A series of 100 frames was captured at approximately 5 Hz, to generate one image set. Such sets were acquired once every minute. Ten sets were generated for each region of the preparation, with at least three regions sampled per preparation (Young *et al.* 2007).

In the first set of experiments, image analysis was performed with Image SXM (http://www.liv.ac.uk/~sdb/ImageSXM/); to correct for lateral movements (particularly the movement generated by contraction), all images were automatically aligned to a template image using the ‘Autoregister’ function of Image SXM and custom-written macros. A region of interest was established which encompassed the portion of a smooth muscle cell that was consistently within the field of view. The fluorescence signal in this region was measured over time throughout the image set. Data were exported to Chart 5 software for measurement of spikes in the Ca^2+^ signal. The threshold for spike detection (based on the amplitude of the first derivative of the fluorescence signal) was manually chosen to match the sensitivity of manual detection for that cell (Zhu *et al.* 2008). The output from Chart 5 was exported to Excel (Microsoft, Redmond, WA, USA) for further analysis, including a calculation of the frequency of spontaneous Ca^2+^ transients and the probability that a field stimulus would evoke a Ca^2+^ transient.

In a second set of experiments, image analysis was performed with an Image J (http://rsb.info.nih.gov/ij/download.html) plug-in written by R. J. Amos (Department of Pharmacology, University of Oxford, Oxford, UK) to detect increases in fluorescence of the Ca^2+^ indicator. All images were automatically aligned to a template image using the ‘Stack reg’ plugin of Image J. In the first frame of the image series, a region of interest was established which encompassed the portion of a smooth muscle cell visible within the confocal plane. The fluorescence signal in this region was measured over time throughout the image set. Data were exported to Chart 5 software to measure calcium spikes and then to Excel for further analysis.

### Statistical analysis

Results are expressed as means ±s.e.m. The area under the response–time curve (AUC) was calculated by the trapezoidal rule with the software GraphPad Prism 4 (GraphPad Software, San Diego, CA, USA). Statistical analysis of the data was performed using Student's *t* test for paired or unpaired samples, or by one-way analysis of variance (ANOVA) followed by Dunnett's *post hoc* test for multiple comparisons. Values of *P* < 0.05 were considered significant. Values of IC_50_ were estimated by linear regression analysis. The number of the strips used corresponds to the number of the animals, unless stated otherwise.

### Drugs: commercial sources and solutions

17β-Oestradiol, carbamylcholine chloride (carbachol), nifedipine, TTX and pluronic F-127 were purchased from Sigma-Aldrich (St Louis, MO, USA). Oregon Green-488 BAPTA-1 AM was purchased from Invitrogen (Paisley, UK) and ICI 182,780 from Tocris Bioscience (Bristol, UK).

Stock solutions (10 mm) were prepared by dissolving 17β-oestradiol and nifedipine in absolute ethanol, kept refrigerated at −20°C and used within 1 week. Stock solutions of ICI 182,780 (10^−2^m) were made by dissolving the drug in DMSO. Aliquots of 10 μm Oregon Green-488 BAPTA-1 AM were obtained by dissolving the powder in 40 μl DMSO and 20% pluronic acid F-127, then adding 460 μl Krebs solution, shielded from light and stored at −20°C. Subsequent dilution, on the day of the experiment, yielded a 10 μm Oregon Green-488 BAPTA-1 AM solution in 1% DMSO and 0.2% pluronic acid. The DMSO and ethanol exerted no significant effects at the maximal concentration used in all the experiments.

## Results

### Effects of E_2_ on detrusor smooth muscle contractility

**Figure 1:**
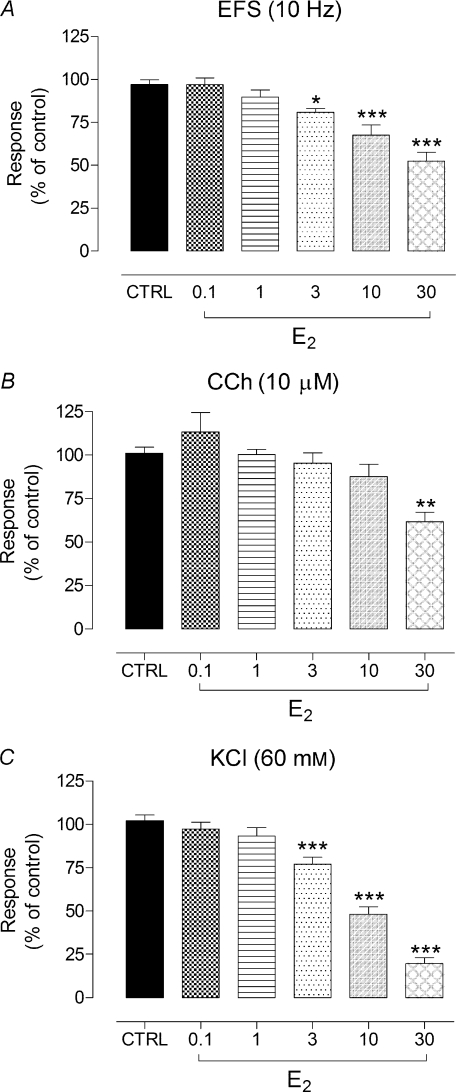
Effect of 17β-oestradiol (E_2_) on the contractions induced by EFS (*A*), CCh (*B*) and KCl (*C*) Results are expressed as a percentage of the control (CTRL) values obtained before applying E_2_ (mean ±s.e.m. of 6 urinary bladders). Statistical analysis was performed using one-way ANOVA followed by Dunnett's *post hoc* test. Significant differences from control groups are indicated; *P < 0.05, **P < 0.01 and ***P < 0.001.

**Figure 2:**
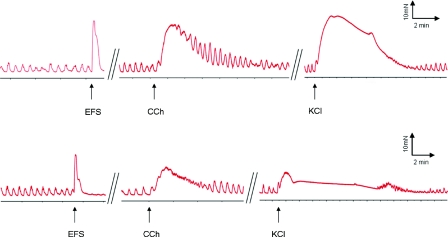
Original traces of contractions induced by EFS, CCh and KCl Representative traces showing responses to EFS (0.05 ms pulse duration, 50 V, 10 Hz, 5 s trains), CCh (10 μm, 10 s application) and KCl (60 mm, 4 min application) from the same detrusor smooth muscle strip in the absence (upper traces) and in the presence of 30 μm E_2_ (lower traces).

**Figure 3:**
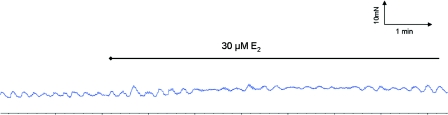
Effect of 30 μm E_2_ on spontaneous activity and baseline of rat detrusor strip Representative trace showing the baseline and the spontaneous activity of rat detrusor strip in the absence and in the presence of 30 μm E_2_.

Detrusor strip responses to EFS (10 Hz), 10 μm carbachol (CCh) and 60 mm KCl in the absence (CTRL) or in the presence of E_2_ at various concentrations are shown in Fig. 1. At 30 μm, E_2_ significantly decreased detrusor contraction induced by all the stimuli applied (see also the original trace in Fig. 2). Both 10 and 3 μm E_2_ significantly decreased EFS- and KCl-induced contractile responses, but at 0.1 and 1 μm E_2_ did not exert any statistically significant effect on detrusor smooth muscle contractility evoked by any of the stimuli. 17β-Oestradiol had no effect on the spontaneous contraction of detrusor strips at any of the concentrations used. At 30 μm, E_2_ significantly increased the resting tone by about 37% (Fig. 3).

Prior incubation with the ER antagonist ICI 182,780 at 1 or 10 μm did not modify the effects of E_2_ on detrusor muscle contractility evoked either by EFS or by the two pharmacological stimuli (CCh and KCl; Table 1).

**Table 1:** Effect of pre-incubation with ICI 182,780 on the contractions induced by electrical field stimulation (EFS), CCh and KCl

	1 μm E_2_	1 μm (E_2_+ ICI 182,780)	10 μm E_2_	10 μm (E_2_+ ICI 182,780)
10 Hz EFS	89.8 ± 6.3	80.4 ± 5.7	67.5 ± 5.9	69.7 ± 4.6
10 μm CCh	100.3 ± 2.9	95.6 ± 2.6	87.6 ± 7.2	82.4 ± 5.2
60 mm KCl	93.2 ± 4.9	88.8 ± 3.4	48.2 ± 4.3	48.5 ± 2.4

Effects of incubation with 1 and 10 μm E_2_, with and without pretreatment with ICI 182,780 (at the same concentrations), on the contractions induced by EFS, CCh and KCl. Results are expressed as means ±s.e.m. (*n*= 6–10). Statistical analysis was performed using Student's *t* test. Any significant difference is detectable.

### Antispasmogenic activity of E_2_ on detrusor smooth muscle strips

In order to gain a better understanding of the inhibitory effect of E_2_ on EFS-induced detrusor contraction, urinary bladder strips, equilibrated in Krebs solution containing 0.5 mm Ca^2+^, were depolarized by Krebs solution containing 60 mm KCl to elicit contractions mediated by Ca^2+^ influx. The tension obtained was 8.3 ± 1.3 mN (*n*= 10 strips from 6 animals), which increased to 20.2 ± 2.7 mN (*n*= 12 strips from 6 animals) at a Ca^2+^ concentration of 5 mm. The Ca^2+^ antagonist activity of E_2_ was assessed by comparing its antispasmogenic activity at the two Ca^2+^ concentrations. 17β-Oestradiol exhibited antispasmogenic activity, since the hormone showed the same behaviour as the well-known Ca^2+^ antagonist nifedipine; its pIC_50_ value significantly decreased as Ca^2+^ concentration increased (Table 2).

**Table 2:** Antispasmogenic effect of nifedipine and 17β-oestradiol (pIC_50_) in detrusor strips superfused with Krebs solution at different Ca^2+^ concentrations and depolarized with 60 mm K^+^

Compound	0.5 mm Ca^2+^	5 mm Ca^2+^
Nifedipine	9.56 ± 0.23	8.77 ± 0.11*
17β-Oestradiol	5.36 ± 0.05	4.86 ± 0.13**

Results are expressed as means ±s.e.m. (*n*= 5) of the pIC_50_. Statistical analysis was performed using Student's *t* test.

* *P* < 0.05

** *P* < 0.01 compared with 0.5 mm Ca^2+^.

### The effect of E_2_ on the amplitude of global Ca^2+^ flashes of male rat detrusor strips

**Figure 4:**
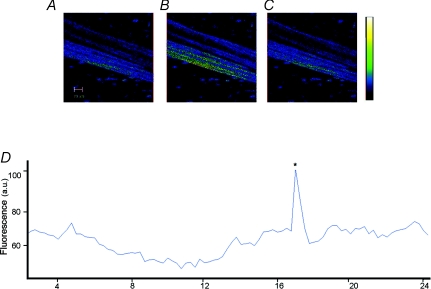
Global Ca^2+^ flashes in urinary bladder smooth muscle strip recorded on confocal microscope A consecutive series of images of smooth muscle cells (running diagonally from upper left to lower right); some muscle cells are closely associated, forming smooth muscle bundles. Images were acquired at 5 frames s^−1^. The images show the smooth muscle prior to (*A*), during (*B*) and after a spontaneous Ca^2+^ global flash (*C*). Note that Ca^2+^ concentration rises rapidly and synchronously in most cells in the field. Below, a typical trace (*D*) showing the fluorescent signal from a portion of a single cell; a single Ca^2+^ flash (*), detected automatically on the basis of a peak in dF/dt, is marked. a.u., arbitrary units.

In order to gain a better understanding of the effect of E_2_ on detrusor smooth muscle contraction, urinary bladder smooth muscle (UBSM) strips were imaged with a laser scanning confocal microscope. Urinary bladder strips, loaded with Oregon Green-488 BAPTA-1 AM, appeared as light green, homogeneous bundles of cells when examined with laser scanning confocal microscopy. In a single field, several smooth muscle cells were often seen lying in parallel (Fig. 4). 17β-Oestradiol at different concentrations (1, 3, 10 and 30 μm) was added to the superfusing Krebs buffer on the confocal microscope stage for 1 h.

**Figure 5:**
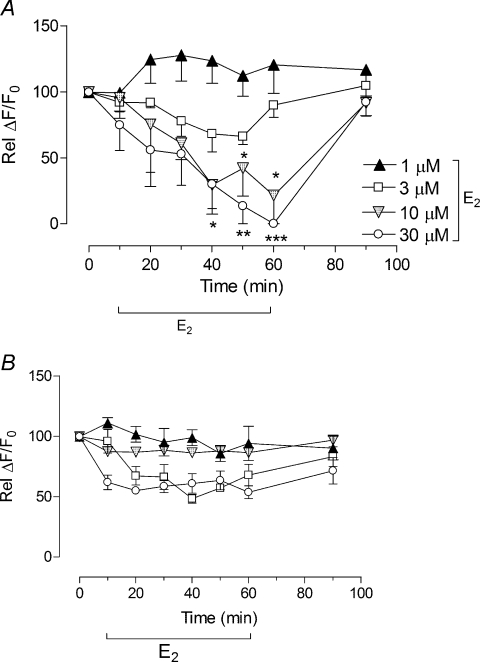
The effect of E_2_ on the amplitude of spontaneous (*A*) and evoked global Ca^2+^ flashes (*B*) Drugs were applied after 30 min of rest and kept for 60 min. The relative change in the fluorescence signal was measured before applying E_2_ (time 0 min, control), during the 60 min of incubation and after 30 min of wash out. Results are expressed as means ±s.e.m. (*n*= 5–8). Statistical analysis was performed using one-way ANOVA followed by Dunnett's *post hoc* test. Significant differences are indicated; *P < 0.05, **P < 0.01 and ***P < 0.001 *versus* control.

In control conditions, spontaneous whole cell Ca^2+^ flashes were observed. Smooth muscle cells displayed repetitive, large and rapid increase in the Ca^2+^ fluorescence that rose almost instantly in a single smooth muscle cell and spread quickly throughout bundle (Fig. 4). The amplitude of such spontaneous whole cell Ca^2+^ flashes was measured, and an increase in the fluorescence of the Ca^2+^ indicator relative to the fluorescence signal at rest (Δ*F*/*F*_0_) was calculated. In the presence of 30 μm E_2_, a gradual decrease of the amplitude of spontaneous Ca^2+^ transient in UBSM strips (Fig. 5*A*) was observed, and after 1 h of treatment the flashes were totally abolished. However, after washing out the hormone, the amplitude of flashes recovered to their initial value. Similar results were obtained in the presence of 10 μm E_2_; after 1 h, a reduction of the amplitude by 20% of the initial value was shown. 17β-Oestradiol at 1 and 3 μm did not have any effect on the amplitude of spontaneous Ca^2+^ flashes.

When UBSM strips were electrically stimulated, Ca^2+^ transients were intermittently evoked. The amplitude of Ca^2+^ transients was less affected than that of spontaneous flashes (not formally tested). In 30 μm E_2_-treated strips the amplitude gradually decreased, reaching approximately 50% of the initial value; however, after washing out the hormone, the amplitude fully recovered. Similarly, in 10 μm E_2_-treated strips the amplitude declined gradually to 60% of the control value. In contrast, at the lowest hormone concentrations tested (1 and 3 μm), the amplitude of evoked Ca^2+^ transients did not differ from the initial amplitude (Fig. 5*B*).

### The effect of E_2_ on the frequency of global Ca^2+^ flashes of male rat detrusor strips

**Figure 6:**
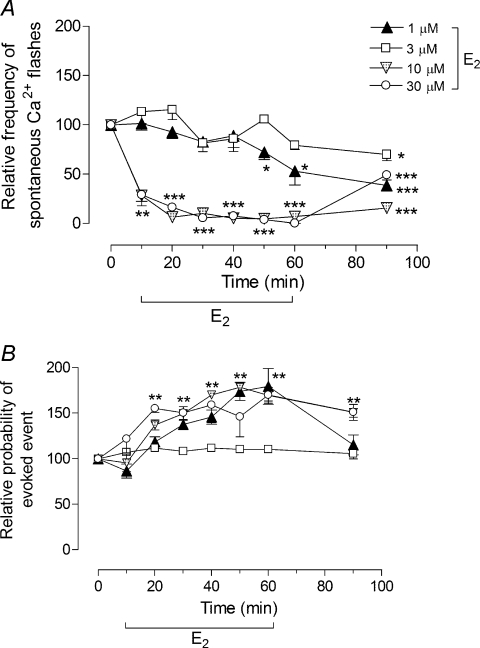
Effect of E_2_ on the frequency of Ca^2+^ transients Drugs were applied after 30 min of rest and kept for 60 min. The frequency of spontaneous Ca^2+^ flashes (*A*) and the probability that EFS evoked a Ca^2+^ transient (*B*) were measured before applying E_2_ (time 0 min, control), during the 60 min of incubation and after 30 min of washing out. Results are expressed as means ±s.e.m. (*n*= 5–8). Statistical analysis was performed using one-way ANOVA followed by Dunnett's *post hoc* test. Significant differences are indicated; *P < 0.05, **P < 0.01 and ***P < 0.001 *versus* control.

17β-Oestradiol significantly reduced the frequency of spontaneous Ca^2+^ transients at all the concentrations tested (Fig. 6*A*). Moreover, the effect of E_2_ on the probability that a field stimulus would evoke a Ca^2+^ transient was also evaluated (Fig. 6*B*). This probability was significantly increased in strips incubated with 10 and 30 μm E_2_, while at 1 and 3 μm E_2_ there was no significant change in the probability that EFS would evoke a Ca^2+^ flash.

### The effect of E_2_ on response to EFS of male rat detrusor strips exposed to 1 h of A-G and 2 h of R

**Figure 7:**
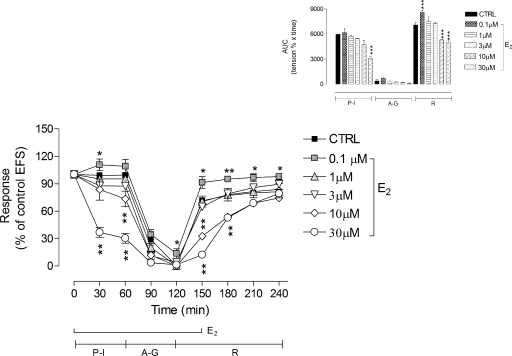
Electrical field stimulation-induced contractile responses of rat detrusor strips subjected to 1 h of A-G and 2 h of R Experiments were carried out in presence or absence (CTRL) of E_2_ at increasing concentrations. Drugs were applied for the first 60 min of the experiment, during A-G and for the first 30 min of R. Results are expressed as means ±s.e.m. (*n*= 4–9). Statistical analysis was performed using one-way analysis of variance (ANOVA) followed by Dunnett's *post hoc* test. Inset shows results expressed as mean of AUC ±s.e.m. (*n*= 4–9), calculated in the P-I, A-G and R phases separately. Statistical analysis was performed using one-way ANOVA followed by Dunnett's *post hoc* test. Significant differences from the control group are indicated; *P < 0.05, **P < 0.01 and ***P < 0.001.

During A-G, the response to EFS in control strips gradually decreased, being abolished within an hour. On the contrary, the response to EFS of 0.1 μm E_2_-treated tissues decreased much more slowly than in control strips, being significantly higher than that of control preparations (*P* < 0.05) at the end of A-G phase. Moreover, 0.1 μm E_2_-treated strips showed a significantly higher recovery during R, compared with control strips. On the contrary, responses to EFS of 10 and 30 μm E_2_-treated strips were significantly lower than those of control strips. At 1 and 3 μm, E_2_ did not exert any significant effect (Fig. 7). Accordingly, the AUC (Fig. 7, inset) of 0.1 μm E_2_-treated strips during the R phase was markedly increased (by 11.4%) compared with the control AUC, while the AUCs of 10 and 30 μm E_2_-treated strips were significantly lower (21.9 and 52.1%, respectively) compared with control tissues. Moreover, E_2_ exerted some significant effects by itself on the response to EFS. In fact, at the highest E_2_ concentrations used (30 μm) the AUC of the pre-incubation phase was significantly lower than that of control strips.

### The effect of E_2_ on response to EFS of male rat detrusor strips exposed to 2 h of A-G and 3 h of R

**Figure 8:**
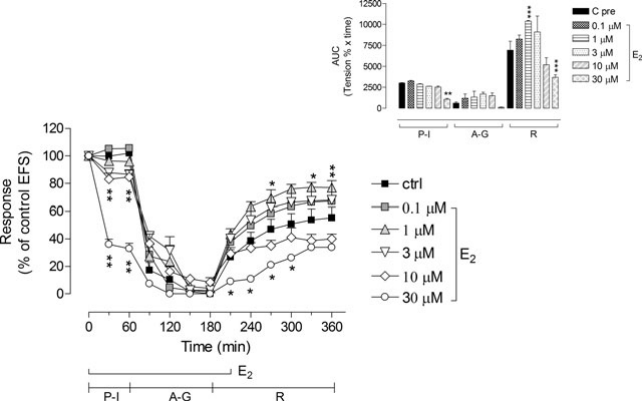
Electrical field stimulation-induced contractile responses of rat detrusor strips subjected to 2 h of A-G and 3 h of R Experiments were carried out in presence or absence (CTRL) of E_2_ at increasing concentrations. Drugs were applied for the first 60 min of the experiment, during A-G and for the first 30 min of R. Results are expressed as means ±s.e.m. (*n*= 4–7). Statistical analysis was performed using one-way ANOVA followed by Dunnett's *post hoc* test. Inset shows results expressed as mean of AUC ±s.e.m. (*n*= 4–7), calculated in the P-I, A-G and R phases separately. Statistical analysis was performed using ANOVA followed by Dunnett's *post hoc* test. Significant differences from the control group are indicated; *P < 0.05, **P < 0.01 and ***P < 0.001.

Figure 8 shows the effects of E_2_ on EFS-induced contractile responses, when the length of exposure to A-G conditions was extended to 120 min. As described in the previous subsection, at the highest E_2_ concentration tested (30 μm), the response to EFS was significantly decreased. However, during the R phase, only 1 μm E_2_-treated strips showed a significantly higher recovery of response to EFS (about 22% higher than that of control strips) while in 30 μm E_2_-treated strips, EFS recovery in the R phase was poor, being around 34% of the initial value.

### Effect of E_2_ on the amplitude of global Ca^2+^ flashes of detrusor strips subjected to 1 h of A-G and 2 h of R

**Figure 9:**
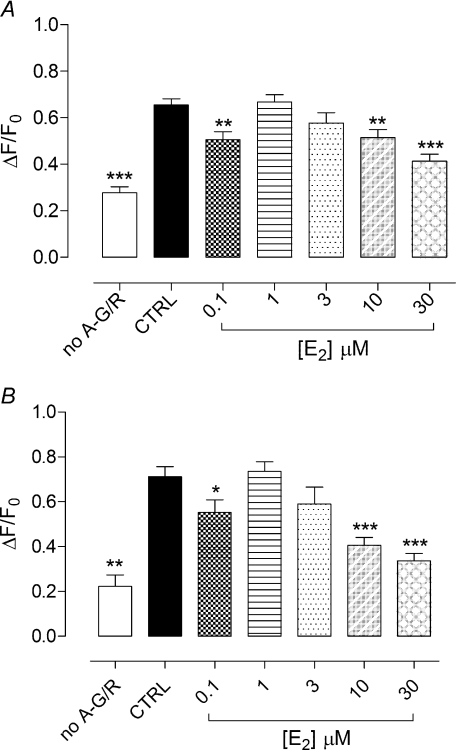
Effect of E_2_ on the amplitude of spontaneous and evoked global Ca^2+^ flashes of strips subjected to 1 h of A-G and 2 h of R Amplitude of spontaneous (*A*) and evoked global Ca^2+^ flashes (*B*) of rat detrusor strips subjected to 60 min of A-G and 120 min of R measured in the absence (CTRL) or presence of E_2_. Results are expressed as means ±s.e.m. (*n*= 6) of Δ*F*/*F*_0_ (first derivative of the fluorescence signal). Statistical analysis was performed using one-way ANOVA followed by Dunnett's *post hoc* test. Significant differences are indicated; **P < 0.01 and ***P < 0.001 *versus* CTRL.

After subjecting tissues to the contraction studies, as shown above, each detrusor strip was imaged with a laser scanning confocal microscope. In the tissues subjected to A-G/R (CTRL) the amplitude of the spontaneous (Fig. 9*A*) and evoked global Ca^2+^ flashes (Fig. 9*B*) was significantly higher than in strips not subjected to A-G/R. At the highest E_2_ concentrations tested (10 and 30 μm), both in spontaneous and evoked whole Ca^2+^ flashes, the amplitude was significantly lower compared with control values.

### Effect of E_2_ on the frequency of global Ca^2+^ flashes of detrusor strips subjected to 1 h of A-G and 2 h of R

**Figure 10:**
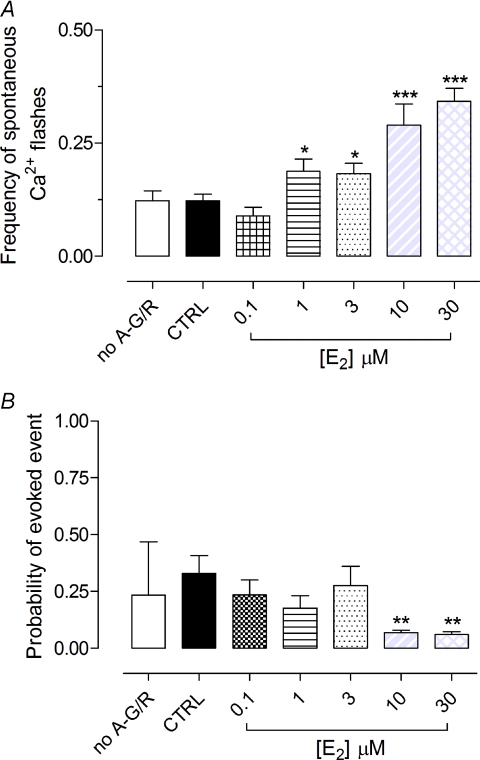
Effect of E_2_ on the frequency of spontaneous and evoked global Ca^2+^ flashes of strips subjected to 1 h of A-G and 2 h of R *A*, frequency of spontaneous Ca^2+^ flashes of detrusor strips subjected to 60 min of A-G and 120 min of R in absence (CTRL) or presence of E_2_. *B*, probability that EFS evoked a Ca^2+^ event. Results are expressed as means ±s.e.m. (*n*= 6). Statistical analysis was performed using one-way ANOVA followed by Dunnett's *post hoc* test. Significant differences are indicated; *P < 0.05, **P < 0.01 and ***P < 0.001 *versus* CTRL.

The frequency of spontaneous Ca^2+^ transients (Fig. 10*A*) and the probability that a field stimulus would evoke a Ca^2+^ transient (Fig. 10*B*) were also determined. There were no significant differences between tissues subjected and not subjected to A-G/R either in spontaneous or in evoked Ca^2+^ flashes. However, in E_2_-treated strips the frequency of whole cell flashes (Fig. 10*A*) increased in a concentration-dependent manner, reaching a significant value above 1 μm E_2_ (*P* < 0.05).

The probability of evoking a flash (Fig. 10*B*), however, was significantly decreased at the highest concentrations of E_2_ used (10 and 30 μm), reaching about 68 and 71% of the control values, respectively.

### Effect of E_2_ on the amplitude and frequency of global Ca^2+^ flashes of detrusor strips subjected to 2 h of A-G and 3 h of R

Using the protocol of 2 h of A-G and 3 h of R, neither consistent effects on spontaneous and evoked Ca^2+^ flashes nor significant changes with E_2_ treatments were seen. Similarly, there were no significant effects either on the frequency of Ca^2+^ transients or on the probability that EFS evoked them at all the concentrations of E_2_ used (data not shown).

## Discussion

In this study, the effect of E_2_ on detrusor smooth muscle contractility was investigated, by using different excitatory stimuli to activate different pathways. To test the possible role of E_2_ as a neuroprotective agent, urinary bladder smooth muscle strips were subjected to A-G/R conditions and the functionality of intrinsic nerves was assessed through EFS. Moreover, since the relaxing effect of E_2_ seems to depend upon the relative contribution of Ca^2+^ influx through voltage-gated Ca^2+^ channels, the effects of E_2_ on global calcium flashes, spontaneous and evoked, were studied.

Numerous epidemiological observations and clinical studies have suggested that oestrogen replacement therapy is associated with beneficial effects on the lower urinary tract in postmenopausal women (Suguita *et al.* 2000; Aikawa *et al.* 2003). However, there are several contradictory reports on the specific effects of oestrogen administration on bladder contractility in animal models (Diep & Constantinou, 1999; Jackson *et al.* 2002). Results from the present study indicate that E_2_ at high concentrations reduces the contractility of male rat urinary bladders in response to either EFS or pharmacological stimuli (CCh and KCl). The effect of E_2_ on the response to CCh, which acts through muscarinic receptors by activating IP_3_-mediated release of calcium from intracellular stores (Mimata *et al.* 1997), was the weakest one compared with the other stimuli. Stimulation with high K^+^, which acts by depolarizing the plasma membranes and triggering calcium entry through L-type calcium channels, elicited the strongest contractions compared with those evoked by EFS and CCh. Taken together, these results suggest that 17β-oestradiol preferentially inhibits pathways requiring depolarization of the muscle cell membrane.

Several lines of evidence argue against the idea that inhibition of contraction induced by E_2_ is mediated by genomic mechanisms involving nuclear ERs, as follows: the concentrations of E_2_ required to reduce smooth muscle contractility are several orders of magnitude higher than those required for genomic activation (McEwen, 1991); the rapid onset of action of E_2_ is also inconsistent with the time course of responses requiring gene transcription; moreover, the selective nuclear ER antagonist did not suppress vascular smooth muscle relaxation by E_2_ (Freay *et al.* 1997). In the present study, the relatively rapid changes in UBSM contractility (contractions were measured after 20 min of incubation with E_2_) and the fact that ICI 182,780 did not inhibit the relaxant effect of E_2_ are also not compatible with the genomic pathway. Furthermore, the lowest concentrations used were in the micromolar range, much higher than physiological plasma levels of E_2_, which are in the nanomolar range. Moreover, Ogata *et al.* (1996) suggested that 17β-oestradiol acts on cell membrane receptors rather than on cytosolic receptors because its action appeared very quickly, taking place through reversible inhibition of voltage-dependent Ca^2+^ channels. In the present study, the Ca^2+^ antagonist effect of E_2_ on rat UBSM is also likely. Firstly, E_2_ caused a concentration-dependent decrease in the KCl-induced contractions, with an IC_50_ for contractile inhibition of 4.0 μm, in agreement with a previous study by Sheldon & Argentieri (1995) on guinea-pig detrusor strips, in which E_2_ had an IC_50_ of 1.7 μm. Secondly, whole cell Ca^2+^ flashes in UBSM, which are diltiazem sensitive and thus require Ca^2+^ influx through voltage-dependent Ca^2+^ channels (Heppner *et al.* 2005), were decreased in amplitude by E_2_ in both a concentration-dependent and a reversible manner. The requirement for smooth muscle action potentials is supported by the observation that action potentials occur spontaneously in UBSM (Heppner *et al.* 1997; Hashitani & Brading, 2003*a*,*b*; Meng *et al.* 2008), and simultaneous recordings of voltage and Ca^2+^ in the guinea-pig UBSM have revealed that each Ca^2+^ transient is associated with an action potential (Hashitani *et al.* 2004). Atropine is unable to affect the spontaneous electrical activity observed in mouse urinary bladder, although such spontaneous depolarizations are abolished when P2X receptors are blocked (Meng *et al.* 2008; Young *et al.* 2008). This suggests that spontaneous ACh release from parasympathetic nerve terminals, coreleased with ATP, is unable to affect the membrane potential. Hence, during brief trains of stimuli the smooth muscle Ca^2+^ transients may well be driven by release of ATP, rather than ACh, from nerve terminals driving smooth muscle action potential and Ca^2+^ influx.

It seems unlikely that an effect of E_2_ on intracellular Ca^2+^ stores can explain the observed inhibition of contraction because: (a) E_2_ reduces the amplitude of the Ca^2+^ flashes, and since these Ca^2+^ flashes depend on the opening of L-type Ca^2+^ channels during smooth muscle action potentials in this tissue (Meng *et al.* 2008; Young *et al.* 2008), inhibition of store release would not be expected to affect the amplitude of Ca^2+^ flashes; and (ii) the ability of E_2_ to reduce KCl-induced contractions also argues against an obligate action on intracellular stores. However, we cannot be sure whether E_2_ decreases electrical excitability, or more directly inhibits L-type Ca^2+^ channels.

In the present work, the slower recovery time of the amplitude of Ca^2+^ events, compared with the effect on their frequency, suggests that the mechanism driving action potential frequency is separate from that determining the amplitude, but that they are both affected by E_2_.

When an obstruction is present or when there is overactivity of the bladder wall, a drop in blood flow and subsequent reduction of substrate and oxygen could occur, resulting in an ischaemic environment within the detrusor (Brading, 1997). The urinary bladder is therefore a good model for studying ischaemic injury. The neuronal damage caused by ischaemic insult plays an important role in the functional defects observed following partial outlet obstruction, nerve terminals being more vulnerable than smooth muscle (Pessina *et al.* 1997). Moreover, previous studies showed that both experimental ischaemia and partial outlet obstruction of the urinary bladder induce similar dysfunction with regard to the contractile responses to EFS (Zhao *et al.* 1997). Therefore, strips pre-incubated with E_2_ were subjected to A-G/R, and whole cell Ca ^2+^ flashes of the same strips were investigated. These cell flashes represent a synchronous increase in the fluorescence of the indicator, hence of Ca^2+^, throughout the visible portion of the smooth muscle cell. In strips subjected to A-G/R, a significant increase in the amplitude of both spontaneous and evoked flashes was observed. At higher concentrations of E_2_, Ca^2+^ antagonist activity seemed to predominate, directly causing a decrease in amplitude of both spontaneous and evoked flashes. Moreover, a A-G/R-induced increase in the frequency of spontaneous Ca^2+^ transients was observed; the cause of this has not been identified. Furthermore, in strips subjected to A-G/R and incubated with high concentrations of E_2_, there was a fall in the probability that a field stimulus evoked a response. 17β-Oestradiol may have decreased transmitter release from the nerves or, alternatively, the high frequency of spontaneous smooth muscle action potentials may have suppressed postjunctional excitability, through, for example, activation of BK channels. Yasay *et al.* (1995) have already demonstrated that E_2_ possesses K^+^ channel opening activity in guinea-pig urinary bladder smooth muscle, activating the Ca^2+^-dependent large-conductance K^+^ channels. Moreover, E_2_, opening BK channels, significantly diminished action potential generation and spontaneous activity, providing negative feedback to limit Ca^2+^ influx (Tanaka *et al.* 2002).

In summary, E_2_, at concentrations of 3 μm and above, suppresses the contractility of urinary bladder smooth muscle to nerve stimulation, consistent with a decrease in the amplitude of the Ca^2+^ response in the smooth muscles cells in conjunction with a decrease in the frequency of spontaneous smooth muscle action potentials. The ability of E_2_ to suppress contraction in control conditions may explain its ability to aid recovery following anoxia–glucopenia, by reducing the metabolic load.

## References

[b1] Aikawa K, Sugino T, Matsumoto S, Chichester P, Whitbeck C, Levin RM (2003). The effect of ovariectomy and estradiol on rabbit bladder smooth muscle contraction and morphology. J Urol.

[b2] Azadzoi KM, Pontari M, Vlachiotis J, Siroky MB (1996). Canine bladder blood flow and oxygenation: changes induced by filling, contraction and outlet obstruction. J Urol.

[b3] Behl C, Holsboer F (1999). The female sex hormone oestrogen as a neuroprotectant. Trends Pharmacol Sci.

[b4] Behl C, Skutella T, Lezoualc’h F, Post A, Widmann M, Newton CJ, Holsboer F (1997). Neuroprotection against oxidative stress by estrogens: structure-activity relationship. Mol Pharmacol.

[b5] Brading AF (1997). A myogenic basis for the overactive bladder. Urology.

[b6] Busby-Whitehead JM, Johnson TM (1998). Urinary incontinence. Clin Geriatr Med.

[b7] Diep N, Constantinou CE (1999). Age dependent response to exogenous estrogen on micturition, contractility and cholinergic receptors of the rat bladder. Life Sci.

[b8] Freay AD, Curtis SW, Korach KS, Rubanyi GM (1997). Mechanism of vascular smooth muscle relaxation by estrogen in depolarized rat and mouse aorta. Role of nuclear estrogen receptor and Ca^2+^ uptake. Circ Res.

[b9] Green PS, Simpkins JW (2000). Neuroprotective effects of estrogen: potential mechanisms of action. Int J Dev Neurosci.

[b10] Greenland JE, Hvistendahl JJ, Andersen H, Jörgensen TM, McMurray G, Cortina-Borja M, Brading AF, Frøkiaer J (2000). The effect of bladder outlet obstruction on tissue oxygen tension and blood flow in the pig bladder. BJU Int.

[b11] Hashitani H, Brading AF (2003a). Electrical properties of detrusor smooth muscles from the pig and human urinary bladder. Br J Pharmacol.

[b12] Hashitani H, Brading AF (2003b). Ionic basis for the regulation of spontaneous excitation in detrusor smooth muscle cells of the guinea-pig urinary bladder. Br J Pharmacol.

[b13] Hashitani H, Brading AF, Suzuki H (2004). Correlation between spontaneous electrical, calcium and mechanical activity in detrusor smooth muscle of the guinea-pig bladder. Br J Pharmacol.

[b14] Hayashi T, Ishikawa T, Yamada K, Kuzuya M, Naito M, Hidaka H, Iguchi A (1994). Biphasic effect of estrogen on neuronal constitutive nitric oxide synthase via Ca^2+^-calmodulin dependent mechanism. Biochem Biophys Res Commun.

[b15] Heppner TJ, Bonev AD, Nelson MT (1997). Ca^2+^-activated K^+^ channels regulate action potential repolarization in urinary bladder smooth muscle. Am J Physiol Cell Physiol.

[b16] Heppner TJ, Bonev AD, Nelson MT (2005). Elementary purinergic Ca^2+^ transients evoked by nerve stimulation in rat urinary bladder smooth muscle. J Physiol.

[b17] Jackson S, James M, Abrams P (2002). The effect of oestradiol on vaginal collagen metabolism in postmenopausal women with genuine stress incontinence. BJOG.

[b18] Kelly MJ, Lagrange AH, Wagner EJ, Rønnekleiv OK (1999). Rapid effects of estrogen to modulate G protein-coupled receptors via activation of protein kinase A and protein kinase C pathways. Steroids.

[b19] Kinn AC, Lindskog M (1988). Estrogens and phenylpropanolamine in combination for stress urinary incontinence in postmenopausal women. Urology.

[b20] Kitazawa T, Hamada E, Kitazawa K, Gaznabi AK (1997). Non-genomic mechanism of 17β-oestradiol-induced inhibition of contraction in mammalian vascular smooth muscle. J Physiol.

[b21] McEwen BS (1991). Non-genomic and genomic effects of steroids on neuronal activity. Trends Pharmacol Sci.

[b22] Matsubara S, Okada H, Shirakawa T, Gotoh A, Kuno T, Kamidono S (2002). Estrogen levels influence beta-3-adrenoceptor-mediated relaxation of the female rat detrusor muscle. Urology.

[b23] Meng E, Young JS, Brading AF (2008). Spontaneous activity of mouse detrusor smooth muscle and the effects of the urothelium. Neurol Urodyn.

[b24] Mimata H, Nomura Y, Emoto A, Latifpour J, Wheeler M, Weiss RM (1997). Muscarinic receptor subtypes and receptor-coupled phosphatidylinositol hydrolysis in rat bladder smooth muscle. Int J Urol.

[b25] Obara K, Bowman PS, Ishida Y, Paul RJ (1997). Effects of hypoxia on [Ca^2+^]_i_, pH_i_ and myosin light chain phosphorylation in guinea-pig taenia caeci. J Physiol.

[b26] Ogata R, Inoue Y, Nakano H, Ito Y, Kitamura K (1996). Oestradiol-induced relaxation of rabbit basilar artery by inhibition of voltage-dependent Ca channels through GTP-binding protein. Br J Pharmacol.

[b27] Pessina F, Kalfin R, Esposito L, Fusi F, Valoti M, Ponticelli F, Sgaragli G (2001). Neuroprotection afforded by some hindered phenols and α-tocopherol in guinea-pig detrusor strips subjected to anoxia-glucopenia and reperfusion-like conditions. Naunyn Schmiedebergs Arch Pharmacol.

[b28] Pessina F, McMurray G, Wiggin A, Brading AF (1997). The effect of anoxia and glucose-free solutions on the contractile response of guinea-pig detrusor strips to intrinsic nerve stimulation and the application of excitatory agonists. J Urol.

[b29] Pessina F, Valeri A, Dragoni S, Valoti M, Sgaragli G (2007). Gender-related neuronal and smooth muscle damage of guinea pig isolated urinary bladder from anoxia-glucopenia and reperfusion injury and its relationship to glycogen content. Neurourol Urodyn.

[b30] Pozzo-Miller LD, Inoue T, Murphy DD (1999). Estradiol increases spine density and NMDA-dependent Ca^2+^ transients in spines of CA1 pyramidal neurons from hippocampal slices. J Neurophysiol.

[b31] Roof RL, Hall ED (2000). Gender differences in acute CNS trauma and stroke: neuroprotective effects of estrogen and progesterone. J Neurotrauma.

[b32] Sheldon JH, Argentieri TM (1995). Acute administration of 17 beta-estradiol inhibits calcium currents in isolated guinea pig detrusor myocytes. J Pharmacol Exp Ther.

[b33] Shimizu S, Bowman PS, Thorne G, Paul RJ (2000). Effects of hypoxia on isometric force, intracellular Ca^2+^, pH, and energetics in porcine coronary artery. Circ Res.

[b34] Stewart WF, Van Rooyen JB, Cundiff GW, Abrams P, Herzog AR, Corey R, Hunt TL, Wein AJ (2003). Prevalence and burden of overactive bladder in the United States. World J Urol.

[b35] Suguita M, Girãio MJ, Simoes MJ, Sartori MG, Baracat ED, Rodrigues de Lima GR (2000). A morphologic and morphometric study of the vesical mucosa and urethra of castrated female rats following estrogen and/or progesterone replacement. Clin Exp Obstet Gynecol.

[b36] Tanaka Y, Horinouchi T, Tanaka H, Shigenobu K, Koike K (2002). BK channels play an important role as a negative feedback mechanism in the regulation of spontaneous rhythmic contraction of urinary bladder smooth muscles. Nippon Yakurigaku Zasshi.

[b37] Yasay GD, Kau ST, Li JH (1995). Mechanoinhibitory effect of estradiol in guinea pig urinary bladder smooth muscles. Pharmacology.

[b38] Young JS, Brain KL, Cunnane TC (2007). The origin of the skewed amplitude distribution of spontaneous excitatory junction potentials in poorly coupled smooth muscle cells. Neuroscience.

[b39] Young JS, Meng E, Cunnane TC, Brain KL (2008). Spontaneous purinergic neurotransmission in the mouse urinary bladder. J Physiol.

[b40] Zhao Y, Levin SS, Wein AJ, Levin RM (1997). Correlation of ischemia/reperfusion or partial outlet obstruction-induced spectrin proteolysis by calpain with contractile dysfunction in rabbit bladder. Urology.

[b41] Zhu HL, Brain KL, Aishima M, Shibata A, Young JS, Sueishi K, Teramoto N (2008). Actions of two main metabolites of propiverine (M-1 and M-2) on voltage-dependent L-type Ca^2+^ currents and Ca^2+^ transients in murine urinary bladder myocytes. J Pharmacol Exp Ther.

